# Supplementary material for: Content-rich biological network constructed by mining PubMed abstracts
Source: BMC Bioinformatics. 2004 Oct 8;5:147. doi: 10.1186/1471-2105-5-147 (PMC528731; doi:10.1186/1471-2105-5-147)
Supplement: Additional File 2 — The original results of the above study (non-essential files are deleted to keep the file size under the limit set by BMC bioinformatics). [file 1471-2105-5-147-S2.bz2 › chilibotAdditionalFile2/dip05/567ID11121055E10931/html/FGF10_FGFR2.html]

 


 **FGF10** and **FGFR2** 
  
Found 47 abstracts in PubMed, retrieved 05.  
 

 What does Google say? 
 PDF only 
| .edu only 

---

**Interactive relationship** (e.g. stimulation, inhibition, etc)

**Neutral relationship**- FGF1, FGF7, FGF9,  **FGF10** , FGF11, and FGF18 were also expressed in the pancreas at varying time points and levels, as well as FGF receptor  [ **FGFR2** ]  forms FGFR1b, FGFR1c, FGFR2b, FGFR2c, FGFR3b, and FGFR4.  Ref: 12666204 Dev Dyn, 2003
- Keratinocyte growth factor receptor  [ **FGFR2** ]  KGFR  [ **FGFR2** ] , also known as fibroblast growth factor receptor FGFR 2  [ **FGFR2** ]  IIIb, is located in many types of epithelial cells and is activated by four known ligands FGF 1, FGF 3, FGF 7 also known as KGF and FGF 10  [ **FGF10** ]  that are predominantly synthesized by mesenchymal cells.  Ref: 12608893 Pathol Int, 2003

**Non-interactive relationship** (e.g. studied together, co-existance, homology, etc.)

- **FGF10**  and FGF receptor  [ **FGFR2** ]  FGFR 2  [ **FGFR2** ]  immunostaining showed a similar epithelial expression pattern.  Ref: 12697038 J Endocrinol, 2003
